# Multi-Layered Filtration Framework for Efficient Detection of Network Attacks Using Machine Learning

**DOI:** 10.3390/s23135829

**Published:** 2023-06-22

**Authors:** Muhammad Arsalan Paracha, Muhammad Sadiq, Junwei Liang, Muhammad Hanif Durad, Muhammad Sheeraz

**Affiliations:** 1 Critical Infrastructure Protection and Malware Analysis Lab, Department of Computer and Information Sciences, Pakistan Institute of Engineering and Applied Sciences, Islamabad 44000, Pakistan; arsalan_18@pieas.edu.pk; 2 Shenzhen Institute of Information Technology, Shenzhen 518109, China; muhammad.sadiq@szu.edu.cn; 3 Department of Computer and Information Sciences, Pakistan Institute of Engineering and Applied Sciences, Islamabad 44000, Pakistan; hanif@pieas.edu.pk (M.H.D.); msheeraz_18@pieas.edu.pk (M.S.)

**Keywords:** intrusion detection system, network attacks, machine learning, network security, security information and event management, CIC-IDS2017, anomaly detection

## Abstract

The advancements and reliance on digital data necessitates dependence on information technology. The growing amount of digital data and their availability over the Internet have given rise to the problem of information security. With the increase in connectivity among devices and networks, maintaining the information security of an asset has now become essential for an organization. Intrusion detection systems (IDS) are widely used in networks for protection against different network attacks. Several machine-learning-based techniques have been used among researchers for the implementation of anomaly-based IDS (AIDS). In the past, the focus primarily remained on the improvement of the accuracy of the system. Efficiency with respect to time is an important aspect of an IDS, which most of the research has thus far somewhat overlooked. For this purpose, we propose a multi-layered filtration framework (MLFF) for feature reduction using a statistical approach. The proposed framework helps reduce the detection time without affecting the accuracy. We use the CIC-IDS2017 dataset for experiments. The proposed framework contains three filters and is connected in sequential order. The accuracy, precision, recall and F1 score are calculated against the selected machine learning models. In addition, the training time and the detection time are also calculated because these parameters are considered important in measuring the performance of a detection system. Generally, decision tree models, random forest methods, and artificial neural networks show better results in the detection of network attacks with minimum detection time.

## 1. Introduction

In today’s digital world, cybersecurity is becoming an essential need for military and government organizations, as well as for small enterprises and even individuals. Threat prevention is the epitome of digital security, which requires threat detection and threat management capabilities [1]. Security information and event management (SIEM) is being implemented by a large number of organizations and becoming a standardized approach to handle information security issues [2]. Due to the recent rise in cyberattacks and the strict security regulations required by governments, organizations have been investing in the security domain [3]. The core of any SIEM solution is the detection capability of the system. Information security experts have developed multiple network intrusion detection tools and techniques for the detection and prevention of evolving network attacks [4].

A computer network is a set of computers connected with each other for resource sharing. Any unauthorized action on the hardware or software of the systems connected with the network is called a network attack. In other words, a network attack is any action that compromises the confidentiality, integrity, or availability of a system. An attack can be classified into one of two types [3]: active attacks and passive attacks. In active attacks, the attacker attempts to alter system resources or affect normal operations. Such attacks can lead to data modification, data loss, data theft, and denial of service. In passive attacks, the attacker attempts to learn important information without affecting the system resources and normal operations. Such attacks can lead to sensitive information being revealed [5].

Denial-of-service (DoS) attacks are the most common type of network attack. In this attack, the attacker tries to engage the system’s resources to deny the service to legitimate users. It is an automated and continuous process that aimst o compromise a system by sending a large number of bogus requests. When these requests are generated from multiple systems, such attacks are known as distributed denial-of-service (DDoS) attacks. In brute-force attacks, the attacker tries multiple combinations to guess the credentials. These attacks are computationally extensive and require huge resources.

Attacks can also be categorized with respect to the origin of the attack, which can be inside attacks or outside attacks [6]. Inside attacks are attacks initiated by an entity within the network or organization and are commonly known as insider attacks. The insider is an authorized user or entity that has access to system resources but uses them in an incorrect way. Outside attacks are attacks initiated by an entity from outside the network or organization and are commonly known as outsider attacks. An outsider is an unauthorized user or entity that performs illegitimate actions.

Broadly, network intrusion detection is categorized into two main types: signature-based detection and anomaly-based detection [7,8]. Static methodology is used in signature-based detection, wherein network traffic is compared with predefined rules, whereas anomaly-based detection is a dynamic method that consists of machine-learning-based models to detect unwanted or malicious behavior over the network [9]. Signature-based approaches directly define the abnormal or malicious behavior in the form of rules. In this case, known threats can be identified very quickly and efficiently. On the other hand, the main objective of anomaly-based approaches is to define the normal or expected behavior so that any variation from normal, or unexpected behavior, can be identified. Therefore, anomaly-based detection techniques can also detect unknown or zero-day attacks [10]. With this clear advantage, anomaly detection would be the preferred methodology among the information security researchers [6,11].

Intrusion detection systems (IDS) serve as essential components in network defense, providing protection against a variety of network attacks. However, the focus of previous research has primarily been on improving the accuracy of IDS while relatively overlooking the crucial aspect of efficiency with respect to time. This oversight limits the effectiveness of IDS in rapidly detecting and responding to network threats.

To address this gap, we propose a multi-layered filtration framework (MLFF) for the efficient detection of network attacks. The primary objective of our research is to minimize detection time without compromising the accuracy of the system. By employing statistical methods for feature reduction, the MLFF systematically reduces the number of features needed, optimizing the efficiency of the intrusion detection process. Through experimentation on the CIC-IDS2017 dataset, we assess the effectiveness of the proposed framework by evaluating its accuracy, precision, recall, and F1 score, in addition to its training time and detection time.

This paper presents an approach to systematically reduce the number of features using statistical methods and proposes a multi-layered framework for feature reduction. The major contributions of this paper are summarized below:

- It provides a multi-layered filtration framework for feature reduction to systematically reduce the number of features using statistical methods.
- It provides a mechanism to effectively reduce the detection time without compromising the accuracy of the detection system.
- It shows the accuracy, precision, recall, F1 score and detection time against selected machine learning models for CIC-IDS2017.

The rest of the paper is organized as follows. Section 2 covers the related work and provides information about available datasets and the evaluation metrics for the detection system. Section 3 provides the methodology and the proposed framework. Section 4 deals with the results and provides a discussion; these aspects are performed through experiments and comparative analysis. Finally, Section 5 covers the conclusions and recommendations for future work.

## 2. Related Work

Research on the detection of network attacks has been conducted using different publicly available datasets. These datasets play a vital role in the validation of the detection approach and are used as benchmarks [7]. The initial work of creating a dataset for an IDS was carried out by DARPA (Defence Advanced Research Project Agency); they generated the KDD98 (Knowledge Discovery and Data Mining) dataset in 1998. This was created by modeling a small US Air Force base network connected to the Internet. It had 41 features that were categorized as normal or abnormal [8]. This dataset plays an important contribution in the research of IDS. However, in [12], the author criticized the accuracy and capability of KDD98 to contemplate realistic environments. Although KDD98 had multiple reported problems, even then, it was being used by the research community [13,14].

Ref. [15] identified numerous issues in the KDD98 dataset, due to which a new dataset NSL-KDD was published in 2009 [16]. The dataset was created by eliminating duplicate records to overcome issues of bias in machine learning models.

Several other IDS datasets have been created. In 2007, a dataset named CAIDA was proposed [17]. This dataset contains network traffic of distributed denial-of-service (DDoS) attacks. This dataset lacks attack diversity. A labeled dataset for flow-based intrusion detection was also proposed in 2009 [18]. The dataset was based on a honeypot deployed over the Internet to maximize exposure to attacks. In 2015, Moustafa and Slay proposed a dataset called UNSW-NB15, which addresses the issue of the unavailability of a network benchmark dataset [19]. The dataset was generated by simulating network attacks and suggesting nine different attack families.

Multiple datasets were reviewed, and in [20], the author proposed 11 characteristics to evaluate a dataset. On the basis of these 11 characteristics, the Canadian Institute for Cybersecurity (CIC) released a dataset called CIC-IDS2017 [21]. The CIC-IDS2017 dataset is now widely being used among security researchers [22,23,24,25,26,27].

Multiple techniques were combined by Yulianto et al. [28] to improve the performance of IDS using the CICIDS-2017 dataset. The hybrid feature selection method was used by Tama et al. [29] and reduced the number of features to 37 with an accuracy of 96.46%.

Gupta et al. [30] suggested that the class imbalance problem can be tackled with the help of ensemble algorithms. Deep neural network, eXtreme gradient boosting, and random forest algorithms were used in three different stages and achieved an accuracy of 92% for the CICIDS-2017 dataset. Doaa et al. [31] also worked on feature reduction along with ensemble learning techniques. The results show 99% accuracy with 30 features from the CICIDS-2017 dataset.

A context-aware feature extraction method was proposed by Shams et al. [32] for convolutional neural networks (CNN); these authors concluded that CNNs showed better results as compared to an ordinary neural network. Birnur et al. [33] proposed an approach using optimal feature selection and finding multivariate outliers for the improvement of the performance of an IDS. The NSL-KDD dataset was used for experiments.

In [34], the author proposed a hybrid optimization scheme to improve the rate of precision in the detection of an intrusion. Qureshi et al. [35] proposed a transfer learning technique to train deep neural networks. The original and extracted features were combined to improve the performance of an intrusion detection system.

Venkatesan et al. [36] suggested a model for intrusion detection and worked on feature selection using a modeling approach. Similarly, in [37,38], the authors worked on reducing the number of network parameters, resulting in decreased time and cost.

In [39], dimensionality reduction was carried out using PCA, and subsequently, SVM was employed for the detection of DDoS attacks in SDN. Ref. [40] also worked on SDN and proposed an allocation-based approach using a multi-criteria decision-making (MCDM) strategy for a multi-domain SDN-enabled IoT network.

In [41], the authors proposed a detection framework based on 16 features for DDoS attack detections. However, this method was not designed to cater to imbalanced data. Yi et al. [42] proposed a multi-objective evolutionary convolutional neural network for an IDS. However, these authors’ results show that the detection performance was affected by the use of very few neurons and layers.

Although much work has been conducted using the available datasets for intrusion detection systems, the major focus remains on the improvement of the accuracy of the detection. In addition to the accuracy, the detection time is also an important factor, especially in the case of AIDS deployed in an inline mode. Our main focus is to propose a framework for feature reduction to effectively reduce the detection time without affecting the accuracy of the system.

### Evaluation Metrics

A confusion matrix is used to evaluate the performance of an intrusion detection system. Table 1 shows a confusion matrix for a binary class classifier.

True positives (*TP*) represent the number of attacks that are correctly predicted as attacks, and true negatives (*TN*) represent the number of normal events or instances that are correctly predicted as normal behavior. False positives (*FP*) represent the number of normal instances that are incorrectly predicted as attacks, and false negatives (*FN*) represent the number of attacks that are incorrectly classified as a normal instance. In a good detection system, *TP* and *TN* should remain high, and *FP* and *FN* should remain low [43].

The accuracy, precision, recall, and F1-score are the performance metrics used to evaluate intrusion detection systems. They are derived from the information given in the confusion matrix and calculated as per the following formulas [7]:

(1) $$Accuracy=\frac{TP+TN}{TP+TN+FP+FN}\times 100$$

(2) $$Precision=\frac{TP}{TP+FP}\times 100$$

(3) $$Recall=\frac{TP}{TP+FN}\times 100$$

(4) $$F1Score=\frac{Precision*Recall}{Precision+Recall}\times 2$$

Accuracy measure how accurate the detection system is at detecting normal and attack traffic. It is the percentage of all correctly predicted instances against all instances. Precision is the accuracy of positive predictions. Recall is the measure of the true-positive rate and is also called the detection rate or sensitivity. F1 score is the harmonic mean of precision and recall. In addition to these performance metrics, we also calculated the training time and the detection time and compared these values against different ML and DL models. In the case of an intrusion detection system, the training time is not considered a very important factor as the model is trained only once. However, the detection time is of prime importance with respect to an intrusion detection system. Both the training time and the detection time are calculated using the formulas below. Table 2 shows a list of notations and their explanations.

(5) TrainingTime=TrEndTime−TrStartTime

(6) DetectionTime=PrEndTime−PrStartTime

## 3. Methodology

The dataset CICIDS2017 was selected as it has been widely used among the research ca ommunity and is also publically available. The dataset was generated on a real network that contained an attacker network and victim network. On the attacker side, four machines were connected, having Kali and Windows 8.1 operating systems. The victim side was protected with a firewall and contained multiple machines with Windows, Linux, and Macintosh operating systems. Multiple services, including domain controller and domain name system (DNS), were also running on the servers so that an attacker could perform real attacks [21]. Almost 50 GB of captured traffic in PCAP files were provided. Along with PCAP files, 8 CSV files were also provided, along with a set of 84 features and a label. The dataset consisted of a total of 15 classes. One of the classes was related to “benign” or normal traffic, and the other 14 corresponded to different “attack” classes. These attack classes were DDoS, Portscan, Bot, Infiltration, Web Attack Brute Force, Web Attack XSS, Web Attack SqlInjection, FTP-Pataor, SSH-Patator, DoS Slowloris, DoS SlowHttp, DoS Hulk, DoS Goldeneye, and Heartbleed.

### 3.1. Proposed Framework Architecture

**Figure 1:**
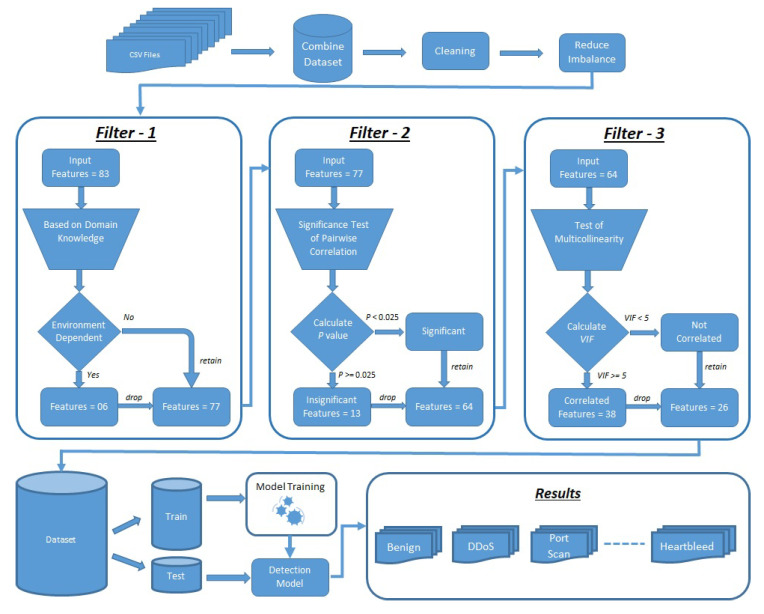
Proposed Framework Architecture.

The proposed multi-layered filtration framework (MLFF) for attack detection is demonstrated in Figure 1. It consists of multiple phases of preprocessing, three layers of feature reduction, the creation of the final dataset, training models on a training dataset, and validating the results on a test dataset.

#### 3.1.1. Merge

As a first step, the 8 CSV files were merged together in order to create a single file that contained the traffic from all classes. This combined dataset was generated from 8 files that were collected from traffic captured from Monday to Friday. A total of 2,520,798 instances were created. Table 3 shows the complete distribution of data among the classes.

#### 3.1.2. Cleaning

After that, the dataset was cleaned from NaN (missing) and infinity values; duplicate values and white spaces were also checked and removed.

#### 3.1.3. Reducing the Imbalance Problem

**Figure 2:**
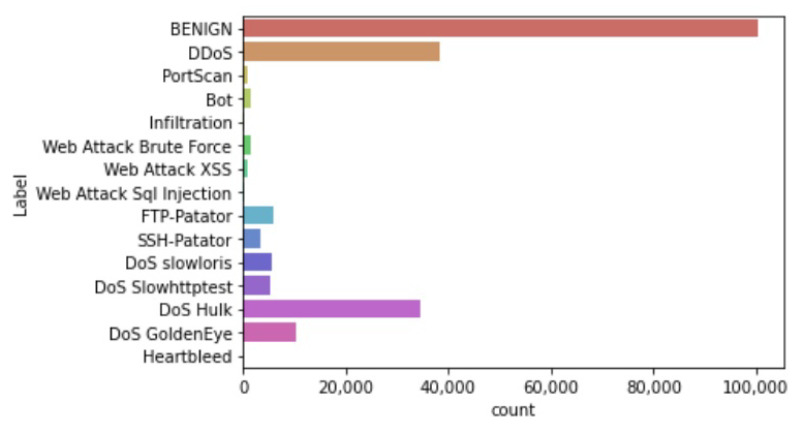
Final dataset distribution.

The results clearly show that the dataset is huge and highly imbalanced. Applying a machine learning model directly may lead to inefficiencies. To overcome the problem of imbalanced data, instances from 4 classes (i.e. Benign, DoS Hulk, DDoS, and Portscan) were removed using the Pandas dataframe.sample method, which returns random samples according to the specified percentage. A total of 31,213 rows were removed because of duplication. After that, a total of 207,908 instances were retained in the dataset. The distribution of these instances among 15 classes is shown in Figure 2.

#### 3.1.4. Filter-1

The first filter was a manual filter in which features were dropped on the basis of domain knowledge. Some features are purely environment-dependent; for example, an IP address can be changed according to the network configurations. Similarly, port numbers can also vary in different scenarios. When the sender wants to communicate with the receiver, it uses a random port number as a source port. Therefore, it is necessary to eliminate such features. If we train our model without removing these features, the model may perform well on test data; however, it will not achieve paramount results in real-world networks.

A total of 83 features and a label are present in the dataset, and of them only 6 features, which were named FlowID, Source IP, Source Port, Destination IP, Destination Port, and Timestamp, were totally dependent on the architecture and the time of the test performed. Therefore, these features were removed from the dataset, and 77 features were left. Table 4 shows the names of the feature dropped in the first layer.

#### 3.1.5. Filter-2

The main objective of our study is to minimize the detection time while maintaining accuracy so that the framework can be used in real networks. In this layer, first, we identified the insignificant features, and then, we dropped these features from the dataset. Therefore, in order to identify the insignificant features, a statistical approach of testing the significance using the *p*-value method was adopted [44]. That is, we wished to test the hypotheses as follows:

(7) $$Null\;Hypothesis\;H_{0}:p=0\;\left(insignificant\right)$$

(8) $$Alternate\;Hypothesis\;H_{a}:p\neq 0\;\left(insignificant\right)$$

The significance of correlation coefficients can be checked with the help of a t-test [45]. In general, we tested the degree of the deviation of the correlation coefficient from zero.

(9) $$t=\frac{r\sqrt{n-2}}{1-r^{2}}$$

Here, *r* is the correlation coefficient calculated from the sample, and n is the sample size. With t and the sample size, we can calculate the p value. If the p value is greater or equal to the significance level, which was set to an alpha equal to 0.025 in our case, we retain the null hypothesis and conclude that the variable is insignificant. If the p value is less than an alpha of 0.025, the null hypothesis is rejected, and we conclude that the variable is significant. Thus, we will not drop it [46].

Because, in our case, the relationship among the features is non-linear, Spearman’s rank correlation coefficient method [47] was used to find the value of *r*.

(10) $$r_{s}=1-\frac{6\sum d^{2}}{n(n^{2}-1)}$$

where,

(11) d=rankX−rankY

The results show that 13 features are insignificant and can be dropped because they do not contribute to the learning of the model. After this test, the number of features was reduced to 64. Table 5 shows the names of the features dropped in the second layer.

#### 3.1.6. Filter-3

The main objective of this filter is to test whether independent variables in a model are correlated among themselves. During this test, we found and removed those independent variables that were highly correlated to avoid the problem of overfitting the model. We selected the variation inflation factor (VIF) method for the detection of multicollinearity [48,49] among independent variables and also calculated the tolerance rate.

(12) $$VIF=\frac{1}{1-R^{2}}$$

(13) $$Tolerance=1-R^{2}$$

Here, $R^{2}$ is the coefficient of determination, which indicates the amount of proportional change in the dependent variable due to the change in the independent variable.

In this case, we selected variables one by one, calculated the VIF against the included variables in a model, ran tests for multicollinearity, and kept the variables that have VIF less than 5. After completing the test iteration, 38 variables were dropped because of their high collinearity with others.

#### 3.1.7. Dataset after Filtration

After passing the parameters through the filtration framework, a new dataset was created with only 26 selected parameters. The dataset was split into training and test data with a ratio of 70/30. The models were trained with training data and evaluated on test data. The training time and the detection time were also recorded against the selected ML models.

## 4. Results and Discussion

This section describes the results obtained after the implementation of a multi-layered feature reduction framework. All the implementation was carried out in Python on Jupyter Notebook (Anaconda3). A Dell OptiPlex 7060 PC with an Intel Core i7-7800 CPU@ 3.40 GHz with 32 GB RAM was used to conduct the experiment.

### 4.1. Selected Parameters

The final selected parameters, variance inflation factor (VIF), and tolerance against each selected feature are shown in Table 6. Among 26 selected parameters, packet length is identified as an important parameter as usually, attack traffic consists of irregular packet length. Flow bytes/s is the packet flow per second. The time between two packets in a flow in a forward and backward direction is also considered an important parameter. Moreover, the number of packets per second in both directions is also selected.

There are different types of flags in a packet header. Each flag has a distinct role in communication. Attackers manipulate the value of these flags to launch attack traffic. The SYN flag is used to initiate a TCP connection. ACK is used to recognize the successful delivery of a packet. The URG flag is used to prioritize the packet. The FIN flag specifies the end of a TCP session. ECE is used to send the congestion indication. The value of these flags and the total number of bytes in both the forward and backward directions sent in an initial window are also selected for the model.

The active mean is the mean time for which the flow remains active, and active std is the standard deviation time before the flow becomes idle. Idle Min is the minimum time a flow remains idle before going active. All these parameters contributed to the efficient detection of network attacks.

### 4.2. Comparison of Results

The main focus of our research is to develop an efficient attack detection system that maintains a high detection rate with a minimum detection time. In this paper, we have calculated the accuracy, precision, recall and F1-score along with the training time and detection time against the selected machine learning models. We compared the results with the findings presented by [24]. The results are also compared with the findings of other researchers and are shown in Table 7.

**Figure 3:**
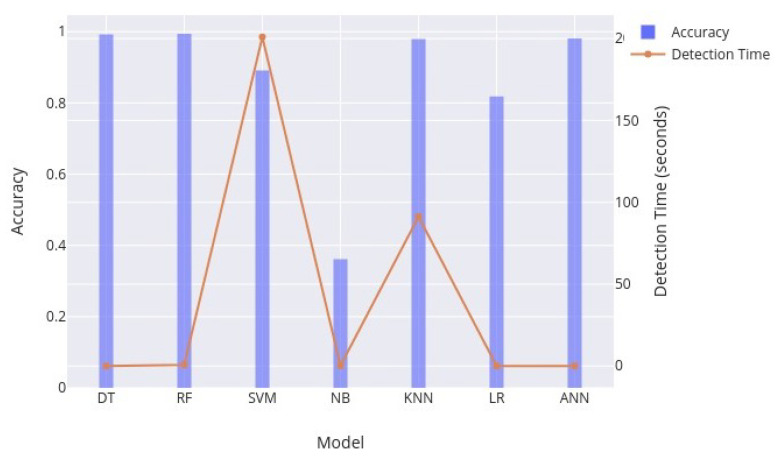
Models with accuracy and detection time of proposed framework.

Figure 3 shows the accuracy and detection time of the proposed framework against selected models. As our main focus is on the reduction of detection time, the results show that in the case of the decision tree (DT) model, the detection time is 0.02 s, as compared to 1.12 s in [24]. Additionally, we still manage to maintain an accuracy of 99.27% as compared to 99.49% in [24]. In addition, random forest (RF) also produces 99.42% accuracy as compared to 99.30% in [24]. The detection time of random forest is calculated as 0.78 s as compared to 6.76 s in [24]. The results of the artificial neural network (ANN) show 98.15% accuracy, which is a little less as compared to 99.28% in [24]. However, the detection time has been significantly reduced, from 48.03 s [24] to 0.03 s.

The results of a K-nearest neighbor (K-NN) classifier show an accuracy of 97.95%, but the detection time is 91.3 s, which makes it impractical for use in an intrusion detection system. Similarly, a support-vector machine (SVM) architecture has a detection time of 201 s, which is also considerably high. The detection time in the case of logistic regression (LR) is very low, at only 0.01 s, but the accuracy is 81.84%. The naïve Bayes (NB) classifier shows an accuracy of only 36.14% with a detection time of 0.21 s. The results clearly show that in our approach, the random forest classifier gives better accuracy with less detection time, and the decision tree classifier gives almost the same accuracy with significantly less detection time.

**Figure 4:**
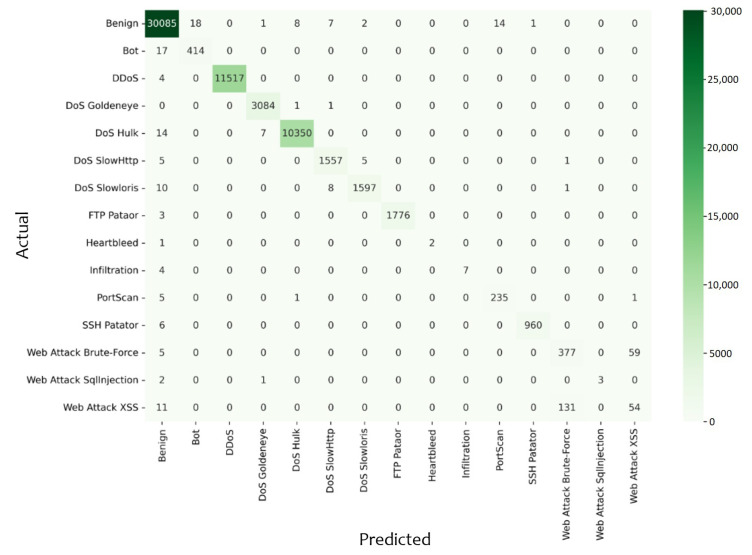
Confusion matrix for random forest model.

As the RF model outperforms all others, the confusion matrix for the random forest model is shown in Figure 4. We can observe that Web Attack Brute-Force and Web Attack XSS have many false positives and false negatives in common. This is due to the high degree of similarity between these attacks.

**Figure 5:**
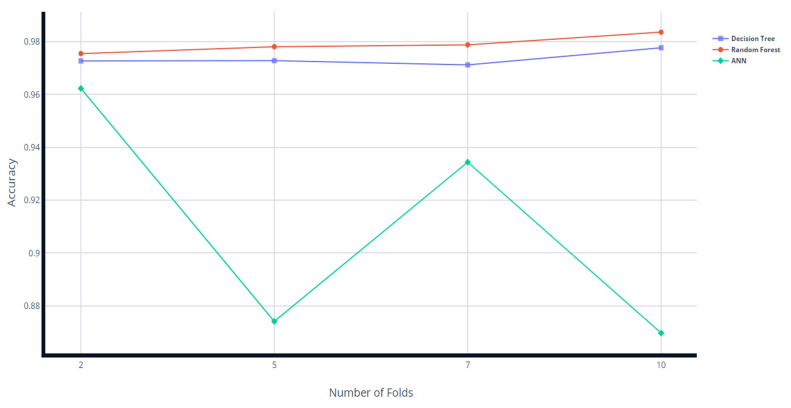
Results of different folds of cross-validation.

We have also calculated the accuracy over 2, 5, 7, and 10 folds of cross-validation for our top 3 models, i.e., decision tree, random forest, and ANN. The results of the average accuracy against each fold are shown in Figure 5. The results show that the decision tree and random forest models perform better with 10-fold cross-validation, and the ANN performs best with 2-fold cross-validation. However, after the analysis of the results of both holdout and cross-validation, it is clear that holdout validation gives better results. The CIC-IDS2017 dataset used in our experiments is a relatively large dataset with a substantial number of samples. This allowed us to split the dataset into separate training and testing sets, ensuring a sufficient amount of data for model training and evaluation. Additionally, our focus was primarily on reducing detection time without compromising accuracy, and holdout validation provided a straightforward and efficient way to assess these metrics. This allowed us to directly compare our results with existing benchmarks and demonstrate the effectiveness of our proposed multi-layered filtration framework (MLFF).

### 4.3. Ablation Study

Our proposed multi-layered filtration framework consists of three essential filters: filter 1 (F1), filter 2 (F2), and filter 3 (F3). In this subsection, we carry out an ablation study to validate their effectiveness by removing or changing the order of these filters. F1 is a mandatory filter and cannot be removed because it is based on environmental parameters, as discussed earlier. However, F2 and F3 can be reordered or removed one by one. Table 8 shows a comparison of the results obtained from this process. For this ablation study, we only selected the random forest ML algorithm because the results in Table 7 clearly show that RF outperforms all other models with respect to accuracy and time.

**Figure 6:**
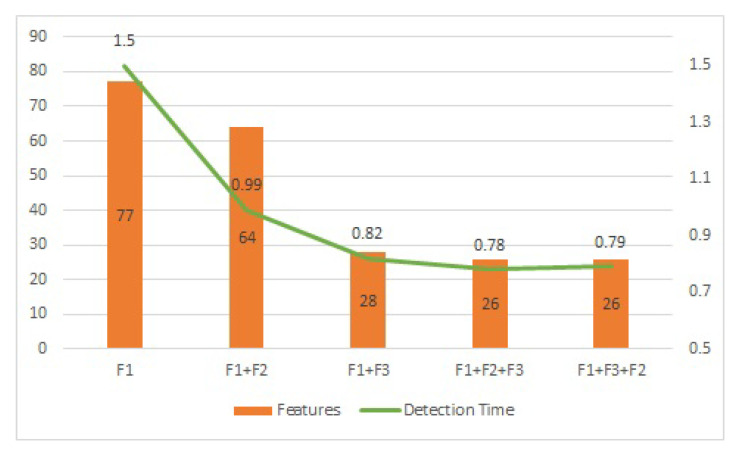
Number of Features and Detection Time against a combination of different filters.

From Figure 6, we can see that each proposed filter plays an important part in reducing the number of features and improving the performance in terms of accuracy and detection time. Furthermore, it can be clearly observed that the best performance is achieved when all three filters are used altogether in the sequence proposed in the multi-layered filtration framework (MLFF).

Using the proposed framework, we managed to maintain a high detection rate while ensuring the minimum detection time by reducing the selected parameters. However, this approach has its own limitations; for example, in the case of high traffic volume, the system cannot be used at an adequate speed. Additionally, this study only focuses on the network attacks used in the CICIDS2017 dataset.

## 5. Conclusions

This paper proposed a multi-layered filtration framework (MLFF) for the efficient detection of network attacks. Using this framework, the number of features were systematically reduced without compromising the performance of the intrusion detection system. The main aim of this paper is to minimize the detection time without affecting the accuracy of the system. The proposed framework contains three filters that are connected in such a way that the output of the first filter becomes the input of the second filter and the output of the second filter becomes the input of the third filter. A total of 26 features were selected out of 83 features. The model was trained using only the selected features, and detection was performed on the test data. The accuracy, precision, recall, and F1-score, along with the training time and detection time, were calculated against the selected machine learning models. The results were compared with the available benchmarks and demonstrated a significant improvement after the implementation of the proposed framework.

Random forest (RF) produced 99.42% accuracy, and the detection time was calculated as 0.78 s. The results clearly showed that random forest outperformed all other models. In addition, the decision tree model and artificial neural networks also performed well. The experiment showed that the detection time was reduced significantly without compromising the accuracy of the system. Because we have managed to reduce the detection time, the proposed framework can be deployed in intrusion detection systems running in real networks.

In order to further enhance the accuracy and timing of the proposed multi-layered filtration framework (MLFF) for the efficient detection of network attacks, future research can focus on leveraging advanced datasets, i.e., cicids2018, to optimize the model. Firstly, expanding the scope of the attack spectrum by incorporating additional attacks from multiple layers would be a valuable direction. By including a wider range of attack types and techniques, the MLFF can improve its ability to detect sophisticated and multi-faceted attacks.

Secondly, future work should explore the utilization of increased computing resources to boost the performance of the MLFF. More powerful hardware, such as high-performance servers or specialized processing units, can significantly accelerate the training and detection processes. This would result in reduced detection times and enable near-real-time analysis of network traffic data. Additionally, the integration of multiple detection frameworks into a centralized security information event management system will improve the security echo system.

## Figures and Tables

**Table 1:** Confusion Matrix.

		Predicted Class	
		Attack	Normal
**Actual** **Class**	Attack	*TP*	*FN*
	Normal	*FP*	*TN*

**Table 2:** List of notations and their explanations.

Notations	Explanaiton
*TP*	True positive
*TN*	True negative
*FP*	False positive
*FN*	False negative
*Ho*	Null hypothesis
*Ha*	Alternate hypothesis
*t*	Calculated value of test statistic
*r*	Correlation coefficient
*n*	Sample size
*d*	Difference between the two ranks of each observation
*R*	Coefficient of determination
*TrstartTime*	Time when training starts
*TrEndTime*	Time when training ends
*PrstartTime*	Time when testing/prediction starts
*PrEndTime*	Time when testing/prediction ends

**Table 3:** Distribution among Classes.

Label	Instances
BENIGN	2,095,057
DoS Hulk	172,846
DDoS	128,014
PortScan	90,694
DoS Goldeneye	10,286
FTP-Pataor	5931
DoS Slowloris	5385
DoS SlowHttp	5228
SSH-Patator	3219
Bot	1948
Web Attack Brute-Force	1470
Web Attack XSS	652
Infiltration	36
Web Attack SqlInjection	21
Heartbleed	11

**Table 4:** Features dropped in the first layer.

S. No.	Feature Name
1	FlowID
2	Source IP
3	Source Port
4	Destination IP
5	Destination Port
6	Timestamp

**Table 5:** Features dropped in the second layer.

S. No	Feature Name
1	Fwd Packet Length Mean
2	Bwd PSH Flags
3	Fwd URG Flags
4	Bwd URG Flags
5	CWE Flag Count
6	Avg Fwd Segment Size
7	Fwd Avg Bytes/Bulk
8	Fwd Avg Packets/Bulk
9	Fwd Avg Bulk Rate
10	Bwd Avg Bytes/Bulk
11	Bwd Avg Packets/Bulk
12	Bwd Avg Bulk Rate
13	Idle Std

**Table 6:** Names of the selected feature.

S. No	Feature Name	VIF	Tolerance
1	Fwd Packet Length Min	1.381	0.724
2	Fwd Packet Length Std	1.145	0.872
3	Bwd Packet Length Min	2.048	0.488
4	Flow Bytes/s	1.078	0.927
5	Flow IAT Min	1.487	0.672
6	Fwd IAT Min	1.927	0.518
7	Bwd IAT Total	3.210	0.311
8	Bwd IAT Std	3.440	0.290
9	Fwd Packets/s	1.161	0.860
10	Bwd Packets/s	1.090	0.917
11	Min Packet Length	2.256	0.443
12	Packet Length Variance	1.350	0.740
13	FIN Flag Count	2.027	0.493
14	SYN Flag Count	1.335	0.748
15	ACK Flag Count	3.256	0.307
16	URG Flag Count	1.731	0.577
17	ECE Flag Count	1.000	0.999
18	Down/Up Ratio	1.625	0.615
19	Subflow Fwd Bytes	1.093	0.914
20	Subflow Bwd Bytes	1.064	0.939
21	Init Win bytes forward	1.916	0.521
22	Init Win bytes backward	1.098	0.910
23	min seg size forward	1.383	0.72
24	Active Mean	1.266	0.789
25	Active Std	1.394	0.717
26	Idle Min	2.522	0.396

**Table 7:** Comparison of results of the proposed framework and previous studies.

Solution	Model	Accuracy	Precision	Recall	F1-Score	Training Time (Seconds)	Detection Time (Seconds)
Proposed	DT	0.9927	0.9927	0.9927	0.9927	1.02	0.02
	RF	0.9942	0.9938	0.9942	0.9939	13.7	0.78
	SVM	0.8911	0.8886	0.8911	0.8812	914	201
	NB	0.3614	0.7070	0.3614	0.3218	0.56	0.21
	KNN	0.9795	0.9795	0.9795	0.9795	0.13	91.3
	LR	0.8184	0.8053	0.8184	0.8041	100	0.01
	ANN	0.9815	0.9824	0.9815	0.9816	84.6	0.03
[24]	DT	0.9949	0.9943	0.9949	0.9942	1.23	1.12
	RF	0.9930	0.9909	0.9930	0.9912	9.38	6.76
	SVM	0.7521	0.9916	0.7521	0.7660	343	33.1
	NB	0.9886	0.9901	0.9886	0.9885	1.07	0.15
	KNN	0.9952	0.9949	0.9952	0.9949	11.13	7.92
	ANN	0.9928	0.9937	0.9928	0.9917	53.78	48.03
[21]	KNN	-	0.96	0.96	0.96	-	-
	RF	-	0.98	0.97	0.97	-	-
	ANN	-	0.98	0.98	0.98	-	-
[25]	CNN + GRU	0.9017	0.9234	0.9124	0.9205	741	17.3
[26]	RF	-	0.9572	0.9458	0.9415	-	
	DBN + SVM	-	0.9774	0.9767	0.9768	-	-
[27]	RF + AutoEncoder	-	-	-	0.9950	-	-
[28]	AdaBoost + SMOTE	0.8147	0.8169	0.9576	0.8817	-	-
[30]	CSE-IDS	0.92	-	-	-	274	0.005
[31]	RF + NB + KNN + SVM	0.997	-	-	-	-	-
[32]	CAFE-CNN	0.992	-	-	-	-	-
[42]	MECNN	0.997	0.991	0.791	83.31	-	-

**Table 8:** Comparison of the proposed framework with different filters.

Filters	Number of Features	Accuracy	Detection Time (s)
F1	77	99.29	1.50
F1 + F2	64	99.30	0.99
F1 + F3	28	99.40	0.82
F1 + F2 + F3	26	99.42	0.78
F1 + F3 + F2	26	99.39	0.79

## Data Availability

The datasets generated during and/or analysed during the current study are available from the corresponding author on reasonable request.

## Abbreviations

The following abbreviations are used in this manuscript:

IDS	Intrusion detection system
AIDS	Anomaly-based intrusion detection system
SIEM	Security information and event management
DARPA	Defence Advanced Research Project Agency
DDOS	Distributed denial of service
PCAP	Packet capture
XSS	Cross-site scripting
FTP	File transfer protocol
SSH	Secure socket shell

## References

[1] Li Y., Liu Q. (2021). A comprehensive review study of cyber-attacks and cyber security; Emerging trends and recent developments. Energy Rep..

[2] Sheeraz M., Paracha M.A., Haque M.U., Durad M.H., Mohsin S.M., Band S.S., Mosavi A. (2023). Effective Security Monitoring Using Efficient SIEM Architecture. Hum.-Centric Comput. Inf. Sci..

[3] Latha S., Prakash S.J. A survey on network attacks and Intrusion detection systems. Proceedings of the 2017 4th International Conference on Advanced Computing and Communication Systems (ICACCS).

[4] Singh R., Srivastav G. Novel Framework for Anomaly Detection Using Machine Learning Technique on CIC-IDS2017 Dataset. Proceedings of the 2021 International Conference on Technological Advancements and Innovations (ICTAI).

[5] Uma M., Padmavathi G. (2013). A Survey on Various Cyber Attacks and their Classification. Int. J. Netw. Secur..

[6] William Stallings L.B. (2015). Computer Security: Principles and Practice.

[7] Thapa N., Liu Z., Kc D.B., Gokaraju B., Roy K. (2020). Comparison of machine learning and deep learning models for network intrusion detection systems. Future Internet.

[8] Khraisat A., Gondal I., Vamplew P., Kamruzzaman J. (2019). Survey of intrusion detection systems: Techniques, datasets and challenges. Cybersecurity.

[9] Aljuhani A. (2021). Machine learning approaches for combating distributed denial of service attacks in modern networking environments. IEEE Access.

[10] Nawaz M., Paracha M.A., Majid A., Durad H. (2020). Attack Detection From Network Traffic using Machine Learning. VFAST Trans. Softw. Eng..

[11] Zhou Y., Cheng G., Jiang S., Dai M. (2020). Building an efficient intrusion detection system based on feature selection and ensemble classifier. Comput. Netw..

[12] Creech G., Hu J. (2013). A semantic approach to host-based intrusion detection systems using contiguousand discontiguous system call patterns. IEEE Trans. Comput..

[13] Ji S.Y., Jeong B.K., Choi S., Jeong D.H. (2016). A multi-level intrusion detection method for abnormal network behaviors. J. Netw. Comput. Appl..

[14] Duque S., bin Omar M.N. (2015). Using data mining algorithms for developing a model for intrusion detection system (IDS). Procedia Comput. Sci..

[15] McHugh J. (2000). Testing intrusion detection systems: A critique of the 1998 and 1999 darpa intrusion detection system evaluations as performed by lincoln laboratory. ACM Trans. Inf. Syst. Secur. (TISSEC).

[16] Tavallaee M., Bagheri E., Lu W., Ghorbani A.A. A detailed analysis of the KDD CUP 99 data set. Proceedings of the 2009 IEEE Symposium on Computational Intelligence for Security and Defense Applications.

[17] Hick P., Aben E., Claffy K., Polterock J. (2007). The CAIDA UCSD “DDoS Attack 2007” Dataset. https://www.caida.org/catalog/datasets/ddos-20070804_dataset/.

[18] Sperotto A., Sadre R., Van Vliet F., Pras A. (2009). A labeled data set for flow-based intrusion detection. Proceedings of the International Workshop on IP Operations and Management.

[19] Moustafa N., Slay J. UNSW-NB15: A comprehensive data set for network intrusion detection systems (UNSW-NB15 network data set). Proceedings of the 2015 military communications and information systems conference (MilCIS).

[20] Gharib A., Sharafaldin I., Lashkari A.H., Ghorbani A.A. An evaluation framework for intrusion detection dataset. Proceedings of the 2016 International Conference on Information Science and Security (ICISS).

[21] Sharafaldin I., Lashkari A.H., Ghorbani A.A. (2018). Toward generating a new intrusion detection dataset and intrusion traffic characterization. Int. Conf. Inf. Syst. Secur. Priv. (Icissp).

[22] Gamage S., Samarabandu J. (2020). Deep learning methods in network intrusion detection: A survey and an objective comparison. J. Netw. Comput. Appl..

[23] Ho S., Al Jufout S., Dajani K., Mozumdar M. (2021). A novel intrusion detection model for detecting known and innovative cyberattacks using convolutional neural network. IEEE Open J. Comput. Soc..

[24] Maseer Z.K., Yusof R., Bahaman N., Mostafa S.A., Foozy C.F.M. (2021). Benchmarking of machine learning for anomaly based intrusion detection systems in the CICIDS2017 dataset. IEEE Access.

[25] Bakhshi T., Ghita B. (2021). Anomaly Detection in Encrypted Internet Traffic Using Hybrid Deep Learning. Secur. Commun. Netw..

[26] Zhang H., Li Y., Lv Z., Sangaiah A.K., Huang T. (2020). A real-time and ubiquitous network attack detection based on deep belief network and support vector machine. IEEE/CAA J. Autom. Sin..

[27] Abdulhammed R., Musafer H., Alessa A., Faezipour M., Abuzneid A. (2019). Features dimensionality reduction approaches for machine learning based network intrusion detection. Electronics.

[28] Yulianto A., Sukarno P., Suwastika N.A. (2019). Improving Adaboost-Based Intrusion Detection System (IDS) Performance on CIC IDS 2017 Dataset.

[29] Tama B.A., Comuzzi M., Rhee K.H. (2019). TSE-IDS: A two-stage classifier ensemble for intelligent anomaly-based intrusion detection system. IEEE Access.

[30] Gupta N., Jindal V., Bedi P. (2022). CSE-IDS: Using cost-sensitive deep learning and ensemble algorithms to handle class imbalance in network-based intrusion detection systems. Comput. Secur..

[31] Mhawi D.N., Aldallal A., Hassan S. (2022). Advanced feature-selection-based hybrid ensemble learning algorithms for network intrusion detection systems. Symmetry.

[32] Shams E.A., Rizaner A., Ulusoy A.H. (2021). A novel context-aware feature extraction method for convolutional neural network-based intrusion detection systems. Neural Comput. Appl..

[33] Uzun B., Ballı S. (2022). A novel method for intrusion detection in computer networks by identifying multivariate outliers and ReliefF feature selection. Neural Comput. Appl..

[34] Velliangiri S., Karthikeyan P. (2020). Hybrid optimization scheme for intrusion detection using considerable feature selection. Neural Comput. Appl..

[35] Qureshi A.S., Khan A., Shamim N., Durad M.H. (2020). Intrusion detection using deep sparse auto-encoder and self-taught learning. Neural Comput. Appl..

[36] Venkatesan S. (2023). Design an Intrusion Detection System based on Feature Selection Using ML Algorithms. Math. Stat. Eng. Appl..

[37] Sadiq M., Shi D. (2022). Attentive occlusion-adaptive deep network for facial landmark detection. Pattern Recognit..

[38] Sadiq M., Shi D., Liang J. (2022). A robust occlusion-adaptive attention-based deep network for facial landmark detection. Appl. Intell..

[39] Ali J., Roh B.h., Lee B., Oh J., Adil M. A Machine Learning Framework for Prevention of Software-Defined Networking controller from DDoS Attacks and dimensionality reduction of big data. Proceedings of the 2020 International Conference on Information and Communication Technology Convergence (ICTC).

[40] Ali J., Jhaveri R.H., Alswailim M., Roh B.h. (2023). ESCALB: An effective slave controller allocation-based load balancing scheme for multi-domain SDN-enabled-IoT networks. J. King Saud Univ.-Comput. Inf. Sci..

[41] Kshirsagar D., Kumar S. (2022). A feature reduction based reflected and exploited DDoS attacks detection system. J. Ambient. Intell. Humaniz. Comput..

[42] Chen Y., Lin Q., Wei W., Ji J., Wong K.C., Coello C.A.C. (2022). Intrusion detection using multi-objective evolutionary convolutional neural network for Internet of Things in Fog computing. Knowl.-Based Syst..

[43] Rosay A., Cheval E., Carlier F., Leroux P. Network Intrusion Detection: A Comprehensive Analysis of CIC-IDS2017. Proceedings of the 8th International Conference on Information Systems Security and Privacy. SCITEPRESS-Science and Technology Publications.

[44] Wonu N., Victor-Edema U.A., Ndimele S.C. (2018). Test of significance of correlation coefficient in science and educational research. Int. J. Math. Stat. Stud..

[45] Keysers C., Gazzola V., Wagenmakers E.J. (2020). Using Bayes factor hypothesis testing in neuroscience to establish evidence of absence. Nat. Neurosci..

[46] Rudolf Freund W.W. (2006). Regression Analysis.

[47] Zar J.H. (1972). Significance testing of the Spearman rank correlation coefficient. J. Am. Stat. Assoc..

[48] Shrestha N. (2020). Detecting multicollinearity in regression analysis. Am. J. Appl. Math. Stat..

[49] Tamura R., Kobayashi K., Takano Y., Miyashiro R., Nakata K., Matsui T. (2019). Mixed integer quadratic optimization formulations for eliminating multicollinearity based on variance inflation factor. J. Glob. Optim..

